# Gibbs Ensemble Monte Carlo Simulation of Fluids in Confinement: Relation between the Differential and Integral Pressures

**DOI:** 10.3390/nano10020293

**Published:** 2020-02-09

**Authors:** Máté Erdős, Olav Galteland, Dick Bedeaux, Signe Kjelstrup, Othonas A. Moultos, Thijs J. H. Vlugt

**Affiliations:** 1 Engineering Thermodynamics, Process & Energy Department, Faculty of Mechanical, Maritime and Materials Engineering, Delft University of Technology, Leeghwaterstraat 39, 2628CB Delft, The Netherlands; m.erdos-2@tudelft.nl (M.E.); o.moultos@tudelft.nl (O.A.M.); 2 PoreLab, Department of Chemistry, Norwegian University of Science and Technology, 7031 Trondheim, Norway; olav.galteland@ntnu.no (O.G.); dick.bedeaux@ntnu.no (D.B.); signe.kjelstrup@ntnu.no (S.K.)

**Keywords:** nanothermodynamics, porous systems, molecular simulation, differential pressure, integral pressure

## Abstract

The accurate description of the behavior of fluids in nanoporous materials is of great importance for numerous industrial applications. Recently, a new approach was reported to calculate the pressure of nanoconfined fluids. In this approach, two different pressures are defined to take into account the smallness of the system: the so-called differential and the integral pressures. Here, the effect of several factors contributing to the confinement of fluids in nanopores are investigated using the definitions of the differential and integral pressures. Monte Carlo (MC) simulations are performed in a variation of the Gibbs ensemble to study the effect of the pore geometry, fluid-wall interactions, and differential pressure of the bulk fluid phase. It is shown that the differential and integral pressure are different for small pores and become equal as the pore size increases. The ratio of the driving forces for mass transport in the bulk and in the confined fluid is also studied. It is found that, for small pore sizes (i.e., <$5\sigma_{\mathrm{fluid}}$), the ratio of the two driving forces considerably deviates from 1.

## 1. Introduction

The widespread application of nanoporous materials in several fields, such as chromatography, membrane separation, catalysis, etc., has lead to a growing interest in the accurate description of the thermodynamic behavior of fluids confined in nanopores [1,2,3,4,5,6]. The pressure of a nanoconfined fluid is one of the most important thermodynamic properties which is needed for an accurate description of the flow rate, diffusion coefficient, and the swelling of the nanoporous material [7,8,9,10]. Various approaches for calculating the pressure of a fluid in a nanopore have been proposed [1,11,12,13]. The main difficulty of the pressure calculation arises from the ambiguous definition of the pressure tensor inside porous materials due to the presence of curved surfaces and confinement effects [7,14,15]. Traditional thermodynamic laws and concepts, such as Gibbs surface dynamics, Kelvin equation, etc., may not be applicable at the nano-scale [16]. In the past decade, several methods were reported using different simulation techniques i.e., classical density functional theory [17,18], equation of state modeling [19], etc., to model the behavior of fluids in confinement.

Recently, Galteland et al. [11] reported a new approach for the calculation of pressure in nanoporous materials using Hill’s thermodynamics for small systems [20]. In this approach, two different pressures are needed to account for confinement effects in nanoporous materials: the differential pressure *P*, and the integral pressure $\hat{p}$. In an ensemble of small systems, the differential pressure times the volume change of the small systems is equal to the work exerted on the surroundings by the volume change. The differential pressure corresponds to the macroscopic pressure and does not depend on the size of the system. The addition of a small system to an ensemble of small systems exerts work on the surroundings which is equal to the integral pressure times the volume of the added small system. The differential and integral pressures are different for small systems and become equal in the thermodynamic limit. For a system with volume *V*, the two pressures are related by [11,20]:

(1) $$P\left(V\right)={\left(\frac{\partial (\hat{p}\left(V\right)V)}{\partial V}\right)}_{T,\mu }=\hat{p}\left(V\right)+V{\left(\frac{\partial (\hat{p}\left(V\right))}{\partial V}\right)}_{T,\mu }.$$

As shown in Equation (1), the two pressures are different only when the integral pressure $\hat{p}$ depends on the volume of the system. Galteland et al. [11] performed equilibrium and non-equilibrium molecular dynamics simulations of Lennard–Jones (LJ) fluids in a face-centered lattice of spherical grains representing a porous medium. Using Hill’s thermodynamics of small systems [20] and the additive property of the grand potential, Galteland et al. [11] defines the compressional energy ($\hat{p}V$) of the representative elementary volume (REV) in the simulations as follows:

(2) $$\hat{p}V={\hat{p}}^{f}V^{f}+{\hat{p}}^{r}V^{r}-{\hat{\gamma }}^{\mathrm{fr}}\omega^{\mathrm{fr}},$$

where $\hat{p}$ is the integral pressure of volume V, ${\hat{p}}^{f}$ and $V^{f}$ are the integral pressure and volume of the fluid, ${\hat{p}}^{r}$ and $V^{r}$ are the integral pressure and volume of the grain particles, and the ${\hat{\gamma }}^{\mathrm{fr}}$ and $\omega^{\mathrm{fr}}$ are the integral surface tension and surface area between the fluid and grain particles. Based on the obtained results, it was concluded that the definition of two pressures is needed to calculate the pressure of the fluid in nanoporous medium.

**Figure 1:**
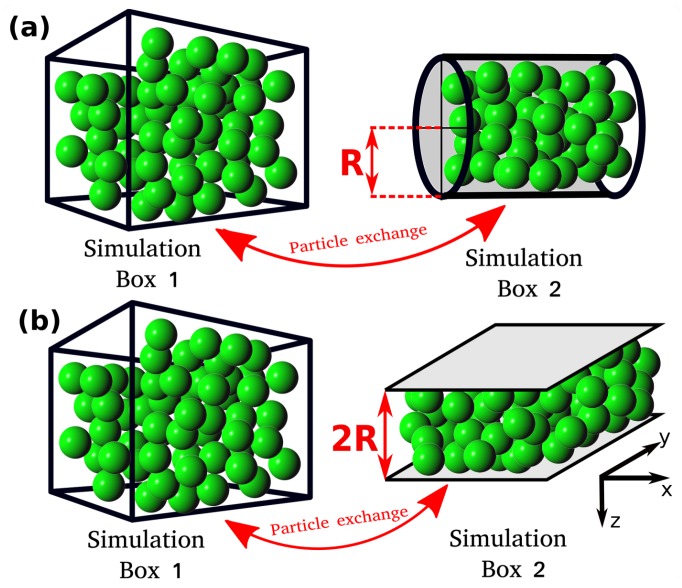
Schematic representation of the Monte Carlo (MC) simulation scheme. Simulation Box 1 represents the bulk fluid with differential pressure *P*. Simulation Box 2 contains the confined fluid with (average) integral pressure $\langle \hat{p}\rangle$. (**a**,**b**) The two investigated systems are shown where the bulk fluid is in equilibrium with the nanoconfined fluid in a cylinder and in a slit pore, respectively. Due to the particle exchange, the chemical potential of the two boxes are equal, but in general $P\neq \langle \hat{p}\rangle$.

In this study, the relation between the differential and integral pressure is investigated by performing Monte Carlo (MC) simulations of LJ fluids. The simulations are carried out in a modified Gibbs ensemble, using two simulation boxes in equilibrium with each other. One box represents the bulk fluid, while the other simulation box represents the nanoconfined system, including walls that interact with the fluid particles, as shown in Figure 1. The effect of confinement on the integral pressure is investigated by considering different fluid-wall interaction strengths and pore geometries, namely, a cylinder and a slit pore. To investigate the relation between the differential and integral pressure, the difference of the two pressures, $P-\langle \hat{p}\rangle$, and the ratio of driving forces for mass transport, $\frac{d\langle \hat{p}\rangle }{dP}$, are computed. In Section 2, the devised ensemble and the equations used to calculate the pressure and energy of the system are presented. In Section 3, a rigorous derivation of the used expression to compute the ratio of driving forces is shown. In Section 4, the results for the different differential pressures and pore geometries are shown. In Section 5, our conclusions are summarized.

## 2. Simulation Details

All MC simulations are carried out using an in-house simulation code. The MC simulations consist of two simulation domains, Simulation Box 1 and Simulation box 2 (see Figure 1). Throughout the manuscript the terms Simulation Box 1 and 2 are used to refer to the two domains, however, simulation domain 2 does not correspond to an actual box. The total number of particles in the system, $N_{T}$, is fixed and particles can be exchanged between the simulation boxes. Box 1 is cubic and has periodic boundary conditions imposed in all directions. Box 1 represents a bulk fluid. The differential pressure, *P*, and temperature, *T*, in Simulation Box 1 are imposed, while the volume, $V_{1}$, and the number of particles, $N_{1}$, can fluctuate. Simulation Box 2 is a cylinder or a slit pore with a fixed volume $V_{2}$. The size of the cylinder and the slit pore are defined by the radius, *R*, and by the distance between the two parallel planes, 2R, respectively (Figure 1). In Box 2, periodic boundary conditions are applied only in the axial direction for the cylinder and in the x and y directions for the slit pore (see Figure 1). Box 2 represents the confined fluid which has an integral pressure $\hat{p}$. In Simulation Box 2, the volume, $V_{2}$, and temperature *T* are imposed, while the number of particles $N_{2}$ can fluctuate by exchanging particles with Box 1. The instantaneous integral pressure fluctuates, and by definition [20] its ensemble average, $\langle \hat{p}\rangle$, will be equal to *P* only for macroscopic systems (R→∞). The ensemble used is a variation of the NPT-Gibbs ensemble [21]. The main difference between the ensemble used in this work and the conventional NPT-Gibbs ensemble is that in our simulations the volume of Box 2 is fixed [21]. Essentially, Box 2 corresponds to the grand canonical ensemble with the reservoir explicitly modeled in Box 1. This computational setup was also used in other studies, i.e., A. Z. Panagiotopoulos et al. [21], P. Bai et al. [22], etc.

**Figure 2:**
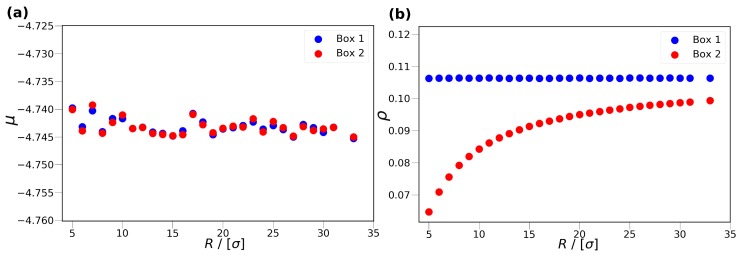
The chemical potential (**a**) and density (**b**) of the bulk, Box 1, and confined fluids, Box 2, as a function of the cylinder radius, *R*, at P=0.2 (in Simulation Box 1). The blue and red colors represent Box 1 and Box 2, respectively. The temperature is fixed at T=2. All values are presented in reduced units. The error bars are smaller than the symbol sizes.

In the MC simulations, three types of trial moves are used: translation, volume change, and particle exchange. The translation and particle exchange trial moves are used in both simulation boxes, while volume change trial moves are only performed in Simulation Box 1. The acceptance rules of the trial moves can be found elsewhere [23]. The particle exchange trial move, ensures that Box 1 and Box 2 are in chemical equilibrium, i.e., the chemical potentials of the two boxes are equal ($\mu_{1}=\mu_{2}$). The chemical potentials of the boxes are defined as the sum of the ideal and excess chemical potentials of the fluid in the respective box ($\mu_{1}=\mu_{1}^{\mathrm{id}}+\mu_{1}^{\mathrm{ex}},\mu_{2}=\mu_{2}^{\mathrm{id}}+\mu_{2}^{\mathrm{ex}}$). The ideal gas chemical potentials ($\mu_{1}^{\mathrm{id}},\mu_{2}^{\mathrm{id}}$) are calculated based on the density and temperature of the fluid. The excess chemical potential ($\mu_{1}^{\mathrm{ex}},\mu_{2}^{\mathrm{ex}}$) can be calculated using different methods, i.e., Widom’s test particle insertion method [23], Contionous Fractional Component Monte Carlo method [24,25], Bennet acceptance ratio method [26], etc. Although the Bennet acceptance ratio method is computationally more efficient than the Widom’s test particle insertion method, the Widom’s test particle method is sufficient for the systems considered in this study. Separate simulations are carried out using different cylinder radii *R*, while imposing P=0.2 (in reduced units) in Box 1. In Figure 2, the chemical potential of Box 1 and Box 2 (Figure 2a), as well as the density of Box 1 and Box 2 (Figure 2b), are shown as a function of the cylinder radius at P=0.2. As can be seen from Figure 2a, the chemical potentials of Box 1 and Box 2 are equal within the uncertainties of the simulations.

In all simulations, the total potential energy, *U*, is calculated using the 12-6 LJ interaction potential:

(3) $$U=U_{\mathrm{fluid}-\mathrm{fluid}}+U_{\mathrm{fluid}-\mathrm{wall}},$$

where $U_{\mathrm{fluid}-\mathrm{fluid}}$ is the potential energy due to interaction between the fluid particles, and $U_{\mathrm{fluid}-\mathrm{wall}}$ represents the potential energy contribution from the interactions between the fluid particles and the wall of Box 2. $U_{\mathrm{fluid}-\mathrm{fluid}}$ in both simulation boxes is calculated according to:

(4) $$U_{\mathrm{fluid}-\mathrm{fluid}}=\left\{\begin{array}{cc} \sum_{i<j}\left[4\epsilon_{\mathrm{fluid}}\left({\left(\frac{\sigma_{\mathrm{fluid}}}{r_{ij}}\right)}^{12}-{\left(\frac{\sigma_{\mathrm{fluid}}}{r_{ij}}\right)}^{6}\right)-U_{\mathrm{shift}}\right] & r_{ij}<2.5\sigma_{\mathrm{fluid}}\\ 0, & \mathrm{otherwise} \end{array}\right.,$$

where $r_{ij}$ is the distance of particle *i* and *j*, $U_{\mathrm{shift}}$ makes the interaction potentially continuous at the cut-off distance ($2.5\sigma_{\mathrm{fluid}}$), and $\epsilon_{\mathrm{fluid}}$, $\sigma_{\mathrm{fluid}}$ are the LJ parameters. In the past, several studies were reported using different types of interaction potentials to model the fluid-solid interactions in confined spaces [22,27,28]. Since the aim of this study is to show the difference between the differential and integral pressures and not to simulate some specific adsorption system, only two types of interaction potentials are considered for the interaction of fluid particles with the wall in Simulation Box 2. In the first case, the wall has only repulsive interactions with the fluid particles. The potential energy contribution of this type is calculated based on the Weeks–Chandler–Andersen potential [29]:

(5) $$U_{\mathrm{fluid}-\mathrm{wall}}=\left\{\begin{array}{cc} \sum_{i=0}^{N_{2}}\left[4\epsilon_{\mathrm{fw}}\left({\left(\frac{\sigma_{\mathrm{fw}}}{r_{wi}}\right)}^{12}-{\left(\frac{\sigma_{\mathrm{fw}}}{r_{wi}}\right)}^{6}\right)+1\right] & r_{wi}<2^{1/6}\sigma_{\mathrm{fw}}\\ 0, & \mathrm{otherwise} \end{array}\right.,$$

where $N_{2}$ is the number of particles in Box 2, $r_{wi}$ is the closest distance of particle *i* from the walls, and $\epsilon_{\mathrm{fw}}$ and $\sigma_{\mathrm{fw}}$ are the LJ parameters for the interaction between the wall and the fluid particles. In the second case, the attractive interactions between the wall and fluid particles are taken into account using the traditional form of the 12-6 LJ interaction potential:

(6) $$U_{\mathrm{fluid}-\mathrm{wall}}=\left\{\begin{array}{cc} \sum_{i=0}^{N_{2}}\left[4\epsilon_{\mathrm{fw}}\left({\left(\frac{\sigma_{\mathrm{fw}}}{r_{wi}}\right)}^{12}-{\left(\frac{\sigma_{\mathrm{fw}}}{r_{wi}}\right)}^{6}\right)-U_{\mathrm{shift}-\mathrm{fw}}\right] & r_{wi}<2.5\sigma_{\mathrm{fw}}\\ 0, & \mathrm{otherwise} \end{array}\right.,$$

where $U_{\mathrm{shift}-\mathrm{fw}}$ makes the interaction potential continuous at the cut-off distance, and $\epsilon_{\mathrm{fw}}$ and $\sigma_{\mathrm{fw}}$ are the LJ parameters.

The expression for calculating the integral pressure in Box 2 is as follows [30]:

(7) $$\hat{p}=\frac{N_{2}}{V_{2}}k_{B}T+{\hat{p}}_{\mathrm{fluid}-\mathrm{fluid}}+{\hat{p}}_{\mathrm{fluid}-\mathrm{wall}},$$

where ${\hat{p}}_{\mathrm{fluid}-\mathrm{fluid}}$ represents the contribution of the fluid-fluid interaction to the integral pressure, $k_{B}$ is the Boltzmann constant, and ${\hat{p}}_{\mathrm{fluid}-\mathrm{wall}}$ represents the contribution of the fluid-wall interaction to the integral pressure. The ${\hat{p}}_{\mathrm{fluid}-\mathrm{fluid}}$, and ${\hat{p}}_{\mathrm{fluid}-\mathrm{wall}}$ terms represent the virial contributions of the integral pressure. The terms are calculated based on the virial theorem [7], i.e., using the derivative of the potential energy function with respect to *r*. The pressure term ${\hat{p}}_{\mathrm{fluid}-\mathrm{fluid}}$ is calculated as follows [7]:

(8) $${\hat{p}}_{\mathrm{fluid}-\mathrm{fluid}}=\frac{48}{3V_{2}}\epsilon_{\mathrm{fluid}}\sum_{i<j}\left({\left(\frac{\sigma_{\mathrm{fluid}}}{r_{ij}}\right)}^{12}-0.5{\left(\frac{\sigma_{\mathrm{fluid}}}{r_{ij}}\right)}^{6}\right).$$

The third term in Equation (7) represents the pressure contribution related to the interactions of the LJ particles with the wall of Box 2. The term ${\hat{p}}_{\mathrm{fluid}-\mathrm{wall}}$ for the repulsive wall potential is derived based on Equation (5) [7]. The following expression is obtained:

(9) $${\hat{p}}_{\mathrm{fluid}-\mathrm{wall}}=\left\{\begin{array}{cc} \sum_{i=1}^{N_{2}}\frac{24}{3V_{2}}\epsilon_{\mathrm{fw}}\left({\left(\frac{\sigma_{\mathrm{fw}}}{r_{wi}}\right)}^{12}-0.5{\left(\frac{\sigma_{\mathrm{fw}}}{r_{wi}}\right)}^{6}\right) & r_{wi}<2^{1/6}\sigma_{\mathrm{fw}}\\ 0, & \mathrm{otherwise} \end{array}\right..$$

The following expression is used to calculate the ${\hat{p}}_{\mathrm{fluid}-\mathrm{wall}}$ when the wall has also attractive interactions with the fluid [7]:

(10) $${\hat{p}}_{\mathrm{fluid}-\mathrm{wall}}=\left\{\begin{array}{cc} \sum_{i=1}^{N_{2}}\frac{24}{3V_{2}}\epsilon_{\mathrm{fw}}\left({\left(\frac{\sigma_{\mathrm{fw}}}{r_{wi}}\right)}^{12}-0.5{\left(\frac{\sigma_{\mathrm{fw}}}{r_{wi}}\right)}^{6}\right) & r_{wi}<2.5\sigma_{\mathrm{fw}}\\ 0, & \mathrm{otherwise} \end{array}\right..$$

By comparing Equations (8)–(10), it can observed that the multiplication factor 48 in Equation (8) is replaced by the factor 24 in Equations (9) and (10). This difference means that only 50 % of the fluid-wall interactions are taken into account at the calculation of the ${\hat{p}}_{\mathrm{fluid}-\mathrm{wall}}$ term [7]. In the MC simulations, Equations (7)–(10) yield instantaneous values from which ensemble averages are computed, i.e., $\langle \hat{p}\rangle$.

In all simulations, the LJ parameters of the fluid particles are $\epsilon_{\mathrm{fluid}}=1$, $\sigma_{\mathrm{fluid}}=1$, and the cut-off radius is $r_{\mathrm{cut}}=2.5\sigma_{\mathrm{fluid}}$ for the fluid-fluid interactions. Regardless of the type of interaction potential used to calculate the interaction of the fluid particles and the wall in Simulation Box 2, the LJ parameter, $\sigma_{\mathrm{fw}}=1$ is used. In the case of the purely repulsive wall potential, the LJ parameter $\epsilon_{\mathrm{fw}}=1$ is used. In case of the attractive wall potential, five different values for the $\epsilon_{\mathrm{fw}}$ LJ parameters are considered: $\epsilon_{\mathrm{fw}}=0.3,0.5,0.7,1.0,1.5$. In this study, all of the reported parameters are in dimensionless units. The LJ interactions are truncated and shifted (i.e., no tail corrections are applied). To avoid phase transitions, the temperature is fixed at T=2 [31,32].

## 3. Theory

**Figure 3:**
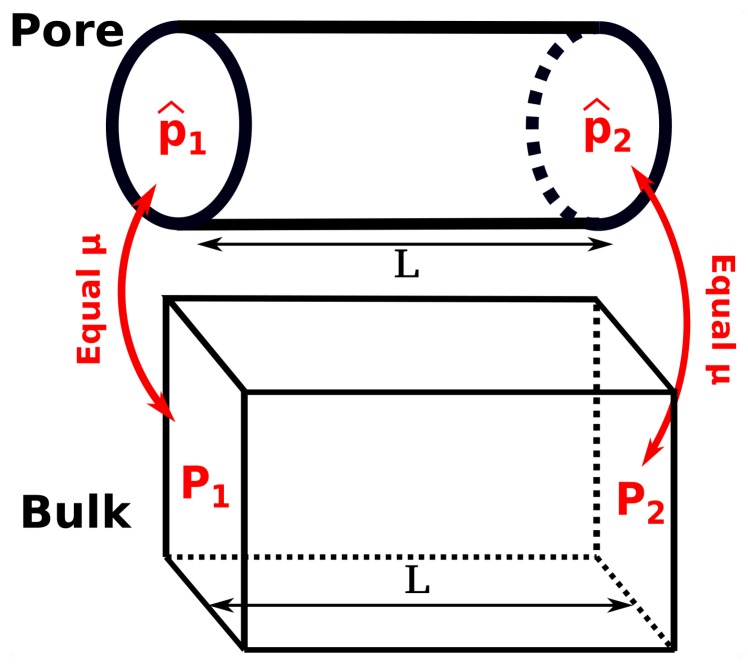
Schematic representation of a bulk fluid in equilibrium with a nanoconfined fluid in a pore. The concept of the ratio of driving forces for mass transport, $\frac{d\langle \hat{p}\rangle }{dP}$, can be introduced based on the definition of ratio of driving forces in the two systems, $\frac{d\langle \hat{p}\rangle }{dL}$ and $\frac{dP}{dL}$.

To investigate the difference between the differential and integral pressures, the ratio $\frac{d\langle \hat{p}\rangle }{dP}$ is calculated. The term $\frac{d\langle \hat{p}\rangle }{dP}$ is the ratio of the pressure gradient for mass transport in the bulk phase, $\frac{dP}{dL}$, and in the confined space $\frac{d\langle \hat{p}\rangle }{dL}$ (see Figure 3). Essentially, the ratio of driving forces, $\frac{d\langle \hat{p}\rangle }{dP}$, equals the ratio of transport coefficients when either *P* or $\hat{p}$ is used as driving force for mass transport in the corresponding transport equation. $\frac{d\langle \hat{p}\rangle }{dP}$ is referred to as the ratio of driving forces throughout this work. One possible approach to compute the ratio of driving forces is to perform simulations at different imposed differential pressures and calculate the difference in the differential and integral pressures. To avoid the necessity of performing several simulations, in this study the ratio of driving forces is calculated based on the fluctuation theory. Using this approach, the ratio of driving forces can be obtained by performing a single simulation.

To obtain an expression for $\frac{d\langle \hat{p}\rangle }{dP}$, the partition function of the system is needed [23]:

(11) $$Q=C\;\sum_{N_{1}=0}^{N_{T}}\frac{V_{2}^{N_{T}-N_{1}}}{N_{1}!\;\left(N_{T}-N_{1}\right)!}\int_{0}^{\infty }dV_{1}\;V_{1}^{N_{1}}\;e^{-\beta PV_{1}}\int dr^{N_{T}}\;e^{-\beta U},$$

where C is a constant, $\beta =\frac{1}{k_{B}T}$, $N_{1}$ is the number of particles in Box 1, $N_{T}$ is total number of particles in the simulation, $V_{1}$ is the volume of Box 1, $V_{2}$ is the volume of Box 2, and *U* is the potential energy. The ensemble average of a thermodynamic property *X* can be obtained using:

(12) $$\langle X\rangle =\frac{\sum_{N_{1}=0}^{N_{T}}\frac{V_{2}^{\left(N_{T}-N_{1}\right)}}{N_{1}!\;\left(N_{T}-N_{1}\right)!}\int_{0}^{\infty }dV_{1}\;V_{1}^{N_{1}}\;e^{-\beta PV_{1}}\int dr^{N_{T}}\;e^{-\beta U}X}{\sum_{N_{1}=0}^{N_{T}}\frac{V_{2}^{\left(N_{T}-N_{1}\right)}}{N_{1}!\;\left(N_{T}-N_{1}\right)!}\int_{0}^{\infty }dV_{1}\;V_{1}^{N_{1}}\;e^{-\beta PV_{1}}\int dr^{N_{T}}\;e^{-\beta U}}.$$

Therefore, to obtain the expression for $\frac{d\langle \hat{p}\rangle }{dP}$, the following relation is used:

(13) $$\frac{d\langle \hat{p}\rangle }{dP}=\frac{d}{dP}\frac{\sum_{N_{1}=0}^{N_{T}}\frac{V_{2}^{\left(N_{T}-N_{1}\right)}}{N_{1}!\;\left(N_{T}-N_{1}\right)!}\int_{0}^{\infty }dV_{1}\;V_{1}^{N_{1}}\;e^{-\beta PV_{1}}\int dr^{N_{T}}\;e^{-\beta U}\hat{p}}{\sum_{N_{1}=0}^{N_{T}}\frac{V_{2}^{\left(N_{T}-N_{1}\right)}}{N_{1}!\;\left(N_{T}-N_{1}\right)!}\int_{0}^{\infty }dV_{1}\;V_{1}^{N_{1}}\;e^{-\beta PV_{1}}\int dr^{N_{T}}\;e^{-\beta U}}.$$

By switching the order of the integration and differentiation, the following expression is obtained:

(14) $$\begin{array}{cc} \; & \frac{d\langle \hat{p}\rangle }{dP}=\\ \; & \frac{\left(\sum_{N_{1}=0}^{N_{T}}\int_{0}^{\infty }dV_{1}\int dr^{N_{T}}\;\frac{V_{2}^{\left(N_{T}-N_{1}\right)}\;V_{1}^{N_{1}}}{N_{1}!\;\left(N_{T}-N_{1}\right)!}\;\left(-\beta \;V_{1}\right)e^{-\beta PV_{1}}\;e^{-\beta U}\hat{p}\right)}{{\left(\sum_{N_{1}=0}^{N_{T}}\int_{0}^{\infty }dV_{1}\int dr^{N_{T}}\;\frac{V_{2}^{\left(N_{T}-N_{1}\right)}\;V_{1}^{N_{1}}}{N_{1}!\;\left(N_{T}-N_{1}\right)!}\;e^{-\beta PV_{1}}\;e^{-\beta U}\right)}^{2}}\\ \; & \times \left(\sum_{N_{1}=0}^{N_{T}}\int_{0}^{\infty }dV_{1}\int dr^{N_{T}}\;\frac{V_{2}^{\left(N_{T}-N_{1}\right)}\;V_{1}^{N_{1}}}{N_{1}!\;\left(N_{T}-N_{1}\right)!}\;e^{-\beta PV_{1}}\;e^{-\beta U}\right)\\ \; & -\frac{\left(\sum_{N_{1}=0}^{N_{T}}\int_{0}^{\infty }dV_{1}\int dr\;\frac{V_{2}^{\left(N_{T}-N_{1}\right)}\;V_{1}^{N_{1}}}{N_{1}!\;\left(N_{T}-N_{1}\right)!}\;\left(-\beta \;V_{1}\right)e^{-\beta PV_{1}}\;e^{-\beta U}\right)}{{\left(\sum_{N_{1}=0}^{N_{T}}\int_{0}^{\infty }dV_{1}\int dr^{N_{T}}\;\frac{V_{2}^{\left(N_{T}-N_{1}\right)}\;V_{1}^{N_{1}}}{N_{1}!\;\left(N_{T}-N_{1}\right)!}\;e^{-\beta PV_{1}}\;e^{-\beta U}\right)}^{2}}\\ \; & \times \left(\sum_{N_{1}=0}^{N_{T}}\int_{0}^{\infty }dV_{1}\int dr^{N_{T}}\;\frac{V_{2}^{\left(N_{T}-N_{1}\right)}\;V_{1}^{N_{1}}}{N_{1}!\;\left(N_{T}-N_{1}\right)!}\;e^{-\beta PV_{1}}\;e^{-\beta U}\hat{p}\right)\\ \; & =\langle -\beta V_{1}\hat{p}\rangle -\left(\langle -\beta V_{1}\rangle \langle \hat{p}\rangle \right)=\beta \left(\langle V_{1}\rangle \langle \hat{p}\rangle -\langle V_{1}\hat{p}\rangle \right)=\frac{\langle V_{1}\rangle \langle \hat{p}\rangle -\langle V_{1}\hat{p}\rangle }{k_{b}T}. \end{array}$$

The final expression in Equation (14) is essentially the cross correlation between $V_{1}$ and $\hat{p}$. In this study, this expression is used to calculate the ratio of driving forces.

## 4. Results and Discussion

In this work, the difference between the differential and integral pressure, $P-\langle \hat{p}\rangle$, and the ratio of driving forces $\frac{d\langle \hat{p}\rangle }{dP}$, are investigated. Two different pore geometries, a cylinder and a slit pore, are studied with varying fluid-wall interaction potentials. The effect of confinement is investigated for gas (ρ≈0.1) and liquid (ρ=0.58,0.8) phases, corresponding to P= 0.2, 2.0, 6.0, respectively.

### 4.1. Difference between the Differential and Integral Pressure

**Figure 4:**
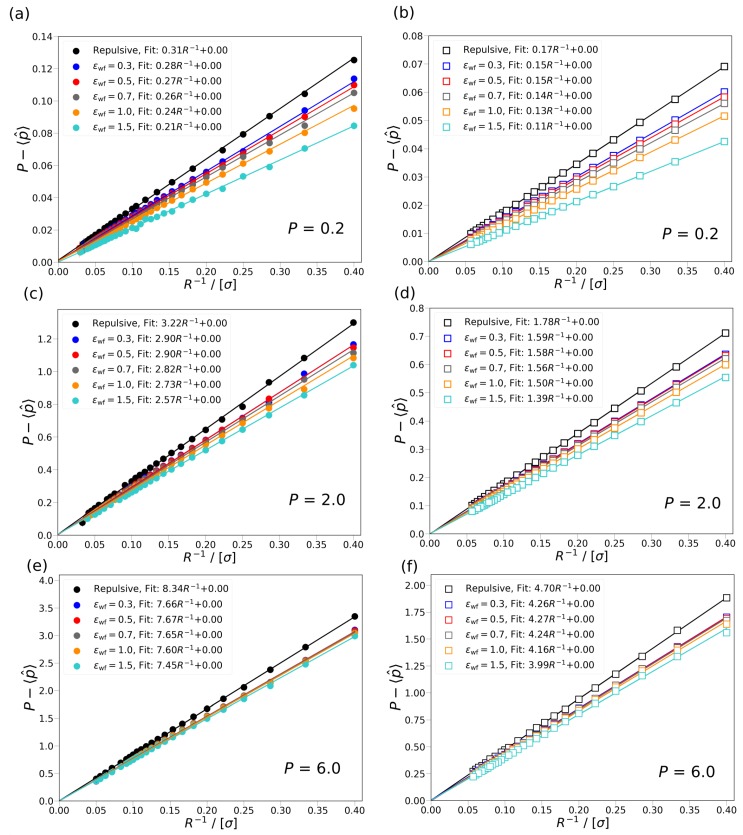
The difference between the differential, *P*, and the ensemble average of integral pressure, $\langle \hat{p}\rangle$, is shown as a function of the inverse radius $R^{-1}$ of Box 2 at P= 0.2, 2.0, and 6.0 for cylindrical and slit pores with fluid-wall interactions. (**a**,**c**,**e**) The pressure difference is shown for cylindrical pores at differential pressure P=0.2, 2.0, and 6.0, respectively. (**b**,**d**,**f**) The pressure difference is shown for slit pores at differential pressure P=0.2, 2.0, and 6.0, respectively. The simulation results are shown with symbols, while the lines are fits to the data points. The equation used for the fitting is $AR^{-1}+B$, where *A* and *B* are constants. The colors denote the different level of attraction between the wall of Box 2 and the fluid, repulsive wall potential (black), $\epsilon_{\mathrm{wf}}=$ 0.3 (blue), 0.5 (red), 0.7 (gray), 1.0 (orange), and 1.5 (cyan). The results for the cylindrical pore are shown with closed circles and for the slit pores with open rectangles. The temperature of both boxes is set to T=2. The average densities of Box 1 are ρ≈ 0.10, 0.58, and 0.8 at P= 0.2, 2.0, and 6.0, respectively.

In Figure 4, the difference between the differential, *P*, and the ensemble average of the integral pressure, $\langle \hat{p}\rangle$, is shown as a function of the inverse radius, $R^{-1}$, of Box 2 for the cylindrical and slit pore cases for the two types of wall potentials. As can be seen in Figure 4, as $R^{-1}$ decreases, the difference between the differential and integral pressure decreases in all cases. The data are fitted to $AR^{-1}$+*B*, where *A* and *B* are constants. The coefficient of determination of the fitted lines is above 0.99 showing that the relation between the $R^{-1}$ and $P-\langle \hat{p}\rangle$ is indeed linear. For large radii (R>30σ), where the fluid in Box 2 behaves like in the bulk, $P-\langle \hat{p}\rangle$ approaches 0, which is also indicated by the fitted lines. In Figure 4a,c,e, the difference between the two pressures are shown for the cylindrical pore. In Figure 4b,d,f, the difference in the pressures are shown for the slit pore. By comparing the magnitude of $P-\langle \hat{p}\rangle$ for the cylindrical and slit pore cases, it can be seen that the pressure difference is larger in the cylindrical pore than in the slit pore. The larger value of $P-\langle \hat{p}\rangle$ for the cylindrical pores can be attributed to the stronger confinement effects compared to the slit pore. The stronger confinement effects are also indicated by the steeper slopes (constant *A* in the fitted lines) of the cylindrical pore compared to the slit pore. It can also be observed that by increasing the interaction strength between the wall of Box 2 and the fluid, $\epsilon_{\mathrm{wf}}$, the difference between the differential and integral pressure at the same pore size decreases. By comparing the calculated values of $P-\langle \hat{p}\rangle$ for P= 0.2 and P= 6.0, it can be seen that the effect of the interaction strength between the wall in Box 2 and the fluid, $\epsilon_{\mathrm{wf}}$, is considerably larger at the lower differential pressure. For example, in the case of the slit pore, the ratio of slopes of the fitted line for $\epsilon_{\mathrm{wf}}=1.0$ and $\epsilon_{\mathrm{wf}}=1.5$ at P=0.2 is 0.88, as shown in Figure 4b, and at P=6.0 is 0.96, as shown in Figure 4f. This can be caused by the different number of particles inside the pore at the two differential pressures. In the case of P=0.2, by increasing the interaction strength, more particles can enter the pore which results in larger integral pressures, while at P=6.0, the pore is practically saturated for all $\epsilon_{\mathrm{wf}}$; therefore, the interaction strength has a lower effect on the integral pressure.

Based on the work of Galteland et al. [11], the slope of the fitted line can also be related to the effective surface tension between the fluid particles and the wall in Box 2, i.e., $P-\langle \hat{p}\rangle ∼\frac{{\hat{\gamma }}_{\mathrm{effective}}^{\mathrm{fr}}}{R}$. The linear relation between $P-\langle \hat{p}\rangle$ and $R^{-1}$ shows that the effective surface tension does not depend on the curvature of the wall. In Figure 4, it can be observed that the effective surface tension decreases as the fluid-wall interactions become more attractive. In Figure 4a–f, it can be seen that the ratio of the effective surface tensions (slopes of the fitted lines) for the same $\epsilon_{\mathrm{wf}}$ with the slit and cylindrical pore is nearly constant. For example, at $\epsilon_{\mathrm{wf}}=0.5$ and P=2.0, the ratio of the slope of the fitted lines for the slit and cylindrical pore is ∼0.54, as shown in Figure 4c,d, and at $\epsilon_{\mathrm{wf}}=0.3$ and P=0.2, the ratio of the two slopes is also ∼0.54, as shown in Figure 4a,b. The ratio of the effective surface tensions between the slit and cylindrical pores are in the range of ∼0.52–0.56 and considered constant since it is within the uncertainties of simulations. The constant ratio of effective surface tension between the slit and cylindrical pore also indicates the larger confinement effects in the cylindrical pore.

### 4.2. Ratio of Driving Forces

**Figure 5:**
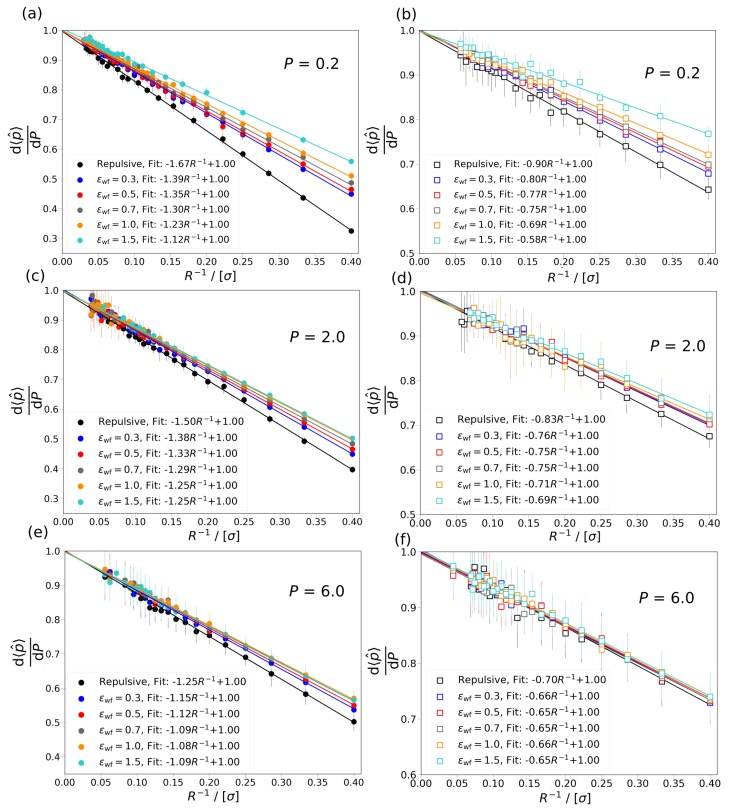
The ratio of driving forces, $\frac{d\langle \hat{p}\rangle }{dP}$, is shown as a function of the inverse radius, $R^{-1}$, of Box 2 at P=0.2,2.0, and 6.0 for the cylindrical and slit pore cases with repulsive and attractive wall potentials. (**a**,**c**,**e**) $\frac{d\langle \hat{p}\rangle }{dP}$ is shown for cylindrical pores at differential pressure P=0.2, 2.0, and 6.0, respectively. (**b**,**d**,**f**) $\frac{d\langle \hat{p}\rangle }{dP}$ is shown for slit pores at differential pressures P=0.2, 2.0, and 6.0, respectively. The simulation results are shown with symbols, while the lines are fits to the data points. The equation used for the fitting is $AR^{-1}+B$, where *A* and *B* are constants. The results for the cylindrical pore are shown with closed circles and for the slit pores with open rectangles. The colors denote the different wall potential used in Box 2, repulsive wall potential (black), $\epsilon_{\mathrm{wf}}=0.3$ (blue), $\epsilon_{\mathrm{wf}}=0.5$ (red), $\epsilon_{\mathrm{wf}}=0.7$ (gray), $\epsilon_{\mathrm{wf}}=1.0$ (orange), and $\epsilon_{\mathrm{wf}}=1.5$ (cyan). The temperature of both boxes is set to T=2. The average densities of Box 1 are ρ≈ 0.10, 0.58, and 0.8 at P= 0.2, 2.0, and 6.0, respectively.

In Figure 5, the ratio of driving forces for mass transport, $\frac{d\langle \hat{p}\rangle }{dP}$, is shown as a function of the inverse radius of Box 2, $R^{-1}$, at three different pressures, P=0.2,2.0,6.0, for both the slit and cylindrical pores. From Figure 5, it can be observed that $\frac{d\langle \hat{p}\rangle }{dP}$ is considerably smaller than 1 for small pore sizes. This means that a change in the differential pressure, *P*, of Box 1 results in a smaller change in the integral pressure, $\langle \hat{p}\rangle$, of Box 2. This difference underlines the effect of the confinement on the pressure of the fluid in nanopores and shows the difference in driving forces in the bulk and confined fluid. As *R* increases, $\frac{d\langle \hat{p}\rangle }{dP}$ approaches 1, i.e., the fluid in the pore behaves more like a bulk fluid. As can be seen in Figure 5, $\frac{d\langle \hat{p}\rangle }{dP}$ is larger for the slit pore than for the cylindrical pore at the same conditions. This means that in case of the slit pore a change in the differential pressure, *P*, results in a larger difference in the integral pressure, $\langle \hat{p}\rangle$, than for the cylindrical pore. The larger change in the integral pressure indicates that the confinement effects are weaker in the slit pore. In Figure 5a,b, it can be observed that by increasing the interaction strength between the wall of the pore and the fluid particles, the ratio of the driving forces decreases. At higher differential pressures the decrease in the ratio of driving forces due to the increasing fluid-wall interaction strength becomes less pronounced, as shown in Figure 5c–f. The smaller influence of the interaction strength, $\epsilon_{\mathrm{fw}}$, on the ratio of driving forces may be caused by the increasing contribution of the fluid-fluid interactions to the integral pressure due to the larger number of fluid particles in the pore at higher differential pressure. In Figure 5a–f, the ratio of driving force is shown for the slit and cylindrical pores at the same differential pressure. It can be seen that the ratio of the slopes of the fitted lines for the same value of $\epsilon_{\mathrm{wf}}$ with the slit and cylindrical pore is constant within the uncertainties of the simulations. The ratio of the slopes is in the range of ∼0.52–0.58, which indicates that the confinement effects in the cylindrical pore are almost twice as strong as in the slit pore.

## 5. Conclusions

In this study, the new approach reported by Galteland et al. [11] is used to investigate the effects of confinement on a fluid in a nanopore by performing MC simulations. The simulations are carried out in a variation of the Gibbs ensemble with two simulation boxes in chemical equilibrium. One of the simulation boxes represents the bulk fluid with differential pressure *P*, and the other a slit or cylindrical pore with repulsive or attractive wall interaction potential. In case of the attractive wall potentials, several scenarios are considered for the strength of the interaction between the wall and the fluid particles. The effect of confinement is investigated for three differential pressures, P= 0.2, 2.0, 6.0, corresponding to gas (ρ≈0.1) and liquid phases (ρ=0.58,0.8). It is concluded that the difference between the differential and integral pressure $P-\langle \hat{p}\rangle$, for all studied cases, approaches 0 when *R*→∞. It is shown that the increase in the interaction strength between the wall and the fluid particles has smaller effect on the difference in the pressures, $P-\langle \hat{p}\rangle$, as the differential pressure increases. Based on the work of Galteland et al. [11], the difference of the differential and integral pressure is related to the effective surface tension between the fluid particles and wall of the pore. It is shown that the effective surface tension does not depend on the curvature of the wall. It is found that by considering a bulk fluid in equilibrium with a cylindrical or slit nanopore, the ratio of driving forces for mass transport in the bulk phase is larger than in the nanopore ($\frac{d\langle \hat{p}\rangle }{dP}<<1$) for small pore sizes. As *R* increases, $\frac{d\langle \hat{p}\rangle }{dP}$ approaches 1, i.e., the fluid in the pore behaves more like a bulk fluid. This clearly shows that the approximation that $\hat{p}\approx P$ does not hold on the nanoscale.

## References

[1] Gubbins K.E., Long Y., Śliwinska Bartkowiak M. (2014). Thermodynamics of confined nano-phases. J. Chem. Thermodyn..

[2] Furukawa H., Cordova K., O’Keeffe M., Yaghi O. (2013). The chemistry and applications of metal-organic frameworks. Science.

[3] Gu Z.Y., Yang C.X., Chang N., Yan X.P. (2012). Metal-organic frameworks for analytical chemistry: From sample collection to chromatographic separation. Acc. Chem. Res..

[4] Yilmaz B., Müller U. (2009). Catalytic applications of zeolites in chemical industry. Top. Catal..

[5] Glaser R., Weitkamp J. (2004). The application of zeolites in catalysis. Springer Ser. Chem. Phys..

[6] Erdős M., de Lange M.F., Kapteijn F., Moultos O.A., Vlugt T.J.H. (2018). In Silico Screening of Metal–Organic Frameworks for Adsorption-Driven Heat Pumps and Chillers. ACS Appl. Mater. Interfaces.

[7] Irving J.H., Kirkwood J.G. (1950). The Statistical Mechanical Theory of Transport Processes. IV. The Equations of Hydrodynamics. J. Chem. Phys..

[8] Férey G., Serre C. (2009). Large breathing effects in three-dimensional porous hybrid matter: Facts, analyses, rules and consequences. Chem. Soc. Rev..

[9] Song H., Yu M., Zhu W., Wu P., Lou Y., Wang Y., Killough J. (2015). Numerical investigation of gas flow rate in shale gas reservoirs with nanoporous media. Int. J. Heat Mass Transf..

[10] Huber P. (2015). Soft matter in hard confinement: Phase transition thermodynamics, structure, texture, diffusion and flow in nanoporous media. J. Phys. Condens. Matter.

[11] Galteland O., Bedeaux D., Hafskjold B., Kjelstrup S. (2019). Pressures Inside a Nano-Porous Medium. The Case of a Single Phase Fluid. Front. Phys..

[12] Todd B.D., Evans D.J., Daivis P.J. (1995). Pressure tensor for inhomogeneous fluids. Phys. Rev. E.

[13] Ikeshoji T., Hafskjold B., Furuholt H. (2003). Molecular-level Calculation Scheme for Pressure in Inhomogeneous Systems of Flat and Spherical Layers. Mol. Simul..

[14] Walton J., Tildesley D., Rowlinson J., Henderson J. (1983). The pressure tensor at the planar surface of a liquid. Mol. Phys..

[15] Blokhuis E.M., Bedeaux D. (1992). Pressure tensor of a spherical interface. J. Chem. Phys..

[16] Wang G.M., Sevick E.M., Mittag E., Searles D.J., Evans D.J. (2002). Experimental Demonstration of Violations of the Second Law of Thermodynamics for Small Systems and Short Time Scales. Phys. Rev. Lett..

[17] Nilson R.H., Griffiths S.K. (2006). Influence of atomistic physics on electro-osmotic flow: An analysis based on density functional theory. J. Chem. Phys..

[18] Lee J.W., Nilson R.H., Templeton J.A., Griffiths S.K., Kung A., Wong B.M. (2012). Comparison of Molecular Dynamics with Classical Density Functional and Poisson–Boltzmann Theories of the Electric Double Layer in Nanochannels. J. Chem. Theory Comput..

[19] Gjennestad M.A., Wilhelmsen Ø. (2020). Thermodynamic stability of droplets, bubbles and thick films in open and closed pores. Fluid Phase Equilibria.

[20] Hill T.L. (1964). Thermodynamics of Small Systems.

[21] Panagiotopoulos A.Z. (1987). Direct determination of phase coexistence properties of fluids by Monte Carlo simulation in a new ensemble. Mol. Phys..

[22] Bai P., Siepmann J.I. (2013). Selective adsorption from dilute solutions: Gibbs ensemble Monte Carlo simulations. Fluid Phase Equilibria.

[23] Frenkel D., Smit B. (2001). Understanding Molecular Simulation.

[24] Shi W., Maginn E.J. (2007). Continuous Fractional Component Monte Carlo: An Adaptive Biasing Method for Open System Atomistic Simulations. J. Chem. Theory Comput..

[25] Poursaeidesfahani A., Torres-Knoop A., Dubbeldam D., Vlugt T.J.H. (2016). Direct Free Energy Calculation in the Continuous Fractional Component Gibbs Ensemble. J. Chem. Theory Comput..

[26] Bennett C.H. (1976). Efficient estimation of free energy differences from Monte Carlo data. J. Comput. Phys..

[27] Stecki J. (1997). Steele (10-4-3) Potential due to a Solid Wall. Langmuir.

[28] Jiménez-Serratos G., Cárdenas H., Müller E.A. (2019). Extension of the effective solid-fluid Steele potential for Mie force fields. Mol. Phys..

[29] Weeks J.D., Chandler D., Andersen H.C. (1971). Role of Repulsive Forces in Determining the Equilibrium Structure of Simple Liquids. J. Chem. Phys..

[30] Allen M.P., Tildesley D.J. (2017). Computer Simulation of Liquids.

[31] Smit B. (1992). Phase diagrams of Lennard–Jones fluids. J. Chem. Phys..

[32] Potoff J.J., Panagiotopoulos A.Z. (1998). Critical point and phase behavior of the pure fluid and a Lennard–Jones mixture. J. Chem. Phys..

